# Sterilization of the Hands and Instruments of Dentists

**Published:** 1893-01

**Authors:** H. A. Smith


### Sterilization of the Hands and Instruments of Dentists.

BY H. A. SMITH, D.D.S.

Read before the Ohio State Dental Society, December, 1892.

In this paper I shall briefly discuss the methods of sterilizing the hands and instruments of the dentist.

In order to fully appreciate the difficulties and exceeding care required to effect perfect sterilization—it may be well to refer to some of the reasons why the dentist should practice it.

I take it that you believe, in a general way, in the germ theory of disease. Referring more especially to some of the diseases of the oral and pharyngeal cavities, you admit that in the production of dental caries micro-organisms play an important part, if, indeed, they are not an essential factor, and in the debris found in deep-seated cavities putrefactive bacteria are always present; that when the pulp cavity is invaded, and the pulp becomes gangrenous it is a very harbor for infectious germs; that pyorrhœa alveolaris—if not a parasitic disease—is in the suppurative stage distinguished by the presence of a large number of pyogenic bacteria; that the disease known as gingivitis is diagnosed by the presence of certain micro-organisms under the free margin of the gums. Admitting the infectious nature of these morbid conditions as well that infections may arise from specific germs of syphilis, diphtheria, and other diseases which manifest their presence upon the tissues of the mouth and pharynx, we are somewhat prepared to appreciate the need of careful sterilization of hands and instruments, following our treatment of these and other communicable diseases.

METHODS.

In the sterilization of dental instruments, the methods usually employed are by the agency of chemical solutions and heat--both moist and dry. For our use the ideal antiseptic solution would be one which acts immediately upon the bacteria without in any way injuring the instrument. Quite a number of antiseptics in solution have from time to time been recommended, but none have proved efficient, unless the instruments are immersed for from ten to thirty minutes.

If these same chemical solutions are heated to near the boiling point they will effect sterilization more rapidly. Dr. Miller, after making quite one thousand tests, concludes that none of the chemical antiseptics at our disposal meet the requirements of a rapid, convenient and absolutely sure mode of sterilizing dental instruments. In reference to the application of heat, Dr. Miller says, "I have found boiling water to accomplish in two minutes as much as the chemical agents ordinarily used in half an hour." Of the employment of dry heat for our sterilizing purposes it may be said that it is effective, but not convenient, and, besides, the temper of the instrument is likely to be taken out, if the proper temperature for sterilization is attained. Coming to the consideration of the employment of moist heat, it may be stated that it is ordinarily more effective than dry heat, and sterilization is accomplished more quickly. The preferable way is to use moist steam heat generated in some good steam sterilizer.

The methods I have described are the methods usually employed in surgical practice, but for our purpose they require too much time. It should be noted that the dental practitioner is often required to sterilize his instruments several times during his operations on the patient. Besides, one patient follows another without delay, and frequently the same instruments are required in the treatment of each case. On the other hand the operations of the general surgeon are usually more formidable, and preparation of his instruments is anticipated, or at least sufficient time, except in emergency cases, may be had for thorough sterilization.

Realizing the importance of a time-saving method of sterilization to the dentist, I have been lead to adopt what may be called sterilization by cremation. It is effected by immersing the infected portion of the instrument in high-proof alcohol, and then igniting the alcohol over a spirit lamp. This may be repeated once or twice without injuring the temper of the instrument. Besides, the repetition gives greater assurance that the adhering bacteria have been thoroughly cremated. If the instrument is held in the hand, the precaution should be observed to hold it at such an angle that the flame shall not come in contact with the hand. If thought desirable, sterilization of such instruments as broaches, scalers, excavators and burrs may be done under the observation of the patient, thereby giving them the assurance that you are using only clean instruments.

Coming to the consideration of sterilization of the dentist's hands, not much, perhaps, need be said. The essentials are hot water, pure soap and a good nail-brush. After a thorough cleansing with these, giving especial attention to the nails, the hands should be carefully dried. Then, just before proceeding with the special operation, the ends of the fingers should be immersed for a moment or two, in the carbolic acid or bi-chloride solution. The expert operator manipulates largely with the ends of his fingers. In handling the mouth, the fingers' ends are brought into contact with diseased tissues. The septic matter in these tissues readily finds its way under the nails; hence the need of special care in cleansing. Some of us, perhaps, can recall instances where negligence in properly sterilizing our fingers has brought about serious trouble, especially in the treatment of root canals. We may sterilize our broach never so carefully, but if we take up cotton with contaminated fingers, and wind it about the broach, and then pass it into a root canal, we have undone all ; we have planted septic bacteria either upon the walls of the canal or have infected the peri-apical tissues. Having found suitable soil, the micro-organisms multiply, and in a short while we have the characteristic inflammation present again. These and similar unfortunate results are frequently attributed to other than the real cause, that is—negligence on the part of the dentist to practice surgical cleanliness. On this point Dr. Miller tersely says: "In the simple act of boring into the pulp chamber we may carry more bacteria into it on our burr than are contained in a whole room full of air. An unclean nerve broach inserted into the root canal may introduce a still greater number."

In dealing with this subject, it should be borne in mind that there is a wide difference between the ordinary cleanliness of our hands and instruments, and what is termed surgical cleanliness. Care in washing with soap and water will accomplish the former. Surgical cleanliness implies that we have to deal with septic or pathogenic bacteria, and that if sterilization is effected, it means the destruction of all the forms of microscopic life that may exist upon our hands or instruments. This can only be accomplished by the use of germicides, or by the adoption of germicidal methods. Both in dentistry and surgery we have a class of practitioners who disparage the value of anti-septic methods. They claim an equal degree of success in their treatment with those who practice the strictest antisepsis. I would not presume to dispute their claims, but I may be permitted the statement that the surgeon or dentist who has any considerable degree of success in his operations without practicing surgical cleanliness, so-called, may at once be classed as an exceedingly clean dentist or surgeon. As a type of this class of surgeons I may name Lawson Tait, of London, of whom it is said that in his operations he carefully avoids anything that resembles Listerism. Notwithstanding all this, he is reputed to be the cleanest surgeon in England. He may use tap-water in his operations, instead of sterilized water, but we may be sure he sees to it that the desired cleanliness is attained. So we have dental practitioners who are successful, and yet they do not believe in the germ theory of disease, neither do they practice technically antiseptic methods. But you may be sure—if you study the methods of such men—that you will find they are exceedingly cleanly, and perhaps unconsciously they are availing themselves of that very method which they disclaim. This class of practitioners always seem to be animated by the sentiment: "Cleanliness is next to godliness"—a beautiful sentiment, indeed, but better adapted to the time of John Wesley than to our time, when, all over the civilized world, scientific research is being made to discover methods whereby infectious diseases may be brought under control and, if possible, ultimately stamped out from the face of the earth. Therefore I would suggest as the more appropriate animating sentiment for our profession: "Cleanliness, plus sterilization, is next to godliness."

